# Review of BCL2 inhibitors for the treatment of Waldenström’s macroglobulinaemia and non-IgM lymphoplasmacytic lymphoma

**DOI:** 10.3389/fonc.2024.1490202

**Published:** 2024-11-04

**Authors:** Oliver Tomkins, Shirley D’Sa

**Affiliations:** UCLH Centre for Waldenström’s Macroglobulinaemia and Related Conditions, Department of Haematology, University College London Hospitals NHS Foundation Trust, London, United Kingdom

**Keywords:** BCL2, Waldenström’s macroglobulinaemia, lymphoplasmacytic lymphoma, venetoclax, sonrotoclax

## Abstract

Lymphoplasmacytic lymphoma (LPL) is a relatively rare form of indolent B-cell non-Hodgkin’s lymphoma, termed Waldenström’s macroglobulinaemia (WM) in the presence of an IgM paraprotein. Although traditionally treated with combination chemoimmunotherapy, the management is evolving in the era of targeted molecular therapies including Bruton’s tyrosine kinase inhibitors (BTKi). However, intolerance and refractoriness to BTKi mean newer agents are required, and the prognosis of so-called quadruple-refractory patients is poor. BCL2 is an anti-apoptotic, pro-survival protein that promotes lymphoma cell survival. Inhibition of BCL2 using first-in-class agent venetoclax has already altered the treatment paradigm in other conditions, including chronic lymphocytic leukaemia (CLL). *In-vivo* inhibition of BCL2 has been shown to lead to apoptosis of LPL/WM cells. Five studies have published results on the use of BCL2 inhibitors in WM to date, including oblimersen sodium, venetoclax, and sonrotoclax. Fixed-duration venetoclax resulted in high response rates, but many patients relapsed following the completion of therapy. The combination of venetoclax with ibrutinib resulted in higher and relatively deep response rates, but unexpected deaths due to ventricular events mean this combination cannot be explored. Two pivotal trials are currently evaluating the use of fixed-duration venetoclax, either in combination with rituximab or pirtobrutinib, whereas another multi-arm study is studying the use of continuous sonrotoclax monotherapy for R/R WM or in fixed-duration combination with Zanubrutinib for treatment-naïve patients. The potential role of BCL2 inhibitors in WM/LPL remains under study, with many hopeful that they may provide an additional chemotherapy-free oral alternative for patients requiring treatment. In an indolent condition with existing effective treatment regimens, including CIT and cBTKi, cost-effectiveness and toxicity profile will be key, although an additional treatment modality for quadruple-refractory patients with limited treatment options is urgently required.

## 1 Introduction

Lymphoplasmacytic lymphoma (LPL) is a relatively rare form of indolent B-cell non-Hodgkin’s lymphoma and a distinct pathophysiological disease entity (1). In the great majority of patients, the lymphoma secretes a monoclonal immunoglobulin M (IgM), and the disease is eponymously termed Waldenström’s macroglobulinaemia (WM). There is an additional recognised subtype, termed non-WM–type LPL that represents approximately 5% of cases and includes those with non-secretory disease, IgG or IgA paraproteins, and technically IgM LPL without bone marrow involvement (2). All are indolent but ultimately incurable conditions. The median age at diagnosis is approximately 70 years, and there is a 2:1 male preponderance. The incidence is estimated at 0.38 per 100,000 persons per year (3).

## 2 Pathophysiology of LPL/WM

WM/LPL arises through the neoplastic proliferation of terminal B cells, which retain the ability to undergo plasmacytic differentiation. This results in an infiltrate of both clonal B and plasma cells in varying proportions. There is a precursor phase, termed monoclonal gammopathy of undetermined significance (MGUS), which is followed by progression to asymptomatic and finally symptomatic LPL/WM. During this process, there is gradual accumulation of LPL with clonal and subclonal molecular evolution. Gradual infiltration in the bone marrow results in cytopaenias, most commonly anemia, but infiltration of other tissues can also occur, including lymph nodes, spleen, bones, and the central nervous system.

Serum IgM concentration appears to correlate with the degree of plasmacytic differentiation of the lymphoma, rather than simply overall disease burden (4). High levels of monoclonal IgM in the plasma result in symptoms of hyperviscosity and acquired von Willebrand syndrome, owing to the immunoglobulin’s large pentameric and hexameric configuration (5). Monoclonal IgM can also cause autoimmune phenomena according to its antigenic target, resulting in complement-mediated destruction, such cold agglutinin syndrome (CAS) and anti-myelin-associated-glycoprotein (MAG) neuropathy. Precipitation of IgM in the form of types I and II cryoglobulin is seen, resulting in cryoglobulinaemic vasculitis (6).

Transformation to a more aggressive, high-grade B-cell lymphoma occurs in up to 4% of patients and has a more adverse prognosis (7).

### 2.1 Disease genomics

Recurring somatic mutations have been identified in LPL cells (8). The activating single-point mutation L265P in the gene *myeloid differentiation primary response 88 (MYD88)* is present in >90% of patients with WM and non-IgM LPL. *MYD88^L265P^* complexes with downstream effectors of B cell receptor signalling, such as IRAK1 and 4, leading to increased activation of the NFκB pathway. In addition, mutated MYD88 causes hyperactivation of haematopoetic cell kinase (HCK), in turn inducing PI3K/AKT, MAPK/ERK, and BTK signalling (9). These pathways drive cell survival and proliferation. Other *MYD88* point mutations have also been described, including S219C, M232T, and S243N (10). MYD88 mutations are absent in patients with IgM multiple myeloma, a wholly separate disease entity characterised by t(11;14) (11).

Additionally, WHIM-like mutations in the gene *C-X-C chemokine receptor type 4 (CXCR4)* are seen in approximately 30%–40% of cases of LPL/WM. These are subclonal and acquired after MYD88-mutations in disease pathogenesis (10, 12). Half are *CXCR4^S338X^*, but >40 frameshift or nonsense mutations are described (13). These mutations result in a CXR4 protein resistant to intracellular inactivation, thereby driving AKT, ERK, and MAPK1/2 pathway signaling, again promoting cellular survival. These mutations are associated with a degree of resistance to BKTi (14), higher serum IgM, and increased incidence of hyperviscosity and acquired von Willebrand syndrome (15, 16).

Other, uncommonly identified somatic mutations include in *AT-rich interactive domain 1A (ARID1A)*, *cluster of differentiation (CD)79B*, and *lysine methyltransferase 2D (KMT2D)* (8, 17, 18). *TP53* aberrations are seen in <10% of patients, associated with resistance to chemotherapy and shorter overall survival (18–21). Most cases of WM also feature multiple chromosomal abnormalities, most frequently deletion 6q, which is associated with progression to symptomatic disease (22, 23).

## 3 Current therapeutic approach

Treatment for WM/LPL is instituted when there are complications or symptoms related to the disease. Many patients can initially be monitored following the diagnosis, in the absence of disease-related symptoms or organ compromise, but the majority come to require therapy (24). The disease is ultimately incurable, but remissions lasting several years following treatment are often seen (25). There is international heterogeneity in the choice of anti-cancer therapy regimen, according to patient age, fitness, comorbidities, disease burden, genomic findings, and local health system reimbursement arrangements.

### 3.1 Combination chemoimmunotherapy

Combination chemoimmunotherapy (CIT) with an anti-CD20 monoclonal antibody (typically rituximab) remains the cornerstone of frontline therapy. Cytotoxic agents, such as purine analogue bendamustine (BR), alkylator cyclophosphamide with dexamethasone (DRC), and the proteasome inhibitor (PI) bortezomib with dexamethasone and cyclophosphamide (B-DRC) are widely used with good efficacy (26, 27). BR results in 2-year overall survival (OS) of 97% and progression-free survival (PFS) 89%, DRC 91%, and 69%, and B-DRC 94 and 81%, respectively (28–30).

### 3.2 Bruton’s tyrosine kinase inhibitors

Targeted agents are dramatically changing the treatment landscape, particularly in the relapsed and refractory (R/R) setting. Following the discovery of *MYD88^L265P^*, the use of targeted covalent BTK inhibitors (cBTKi) was shown to lead to apoptosis of WM cells (31, 32) leading to successful clinical trials (33). cBTKis are now commonly employed at relapse and continued until disease progression. In some healthcare systems, it is also available for frontline use without prior exposure to CIT and may be preferable to CIT in those with significant comorbidities or frailty (27). First-generation cBTKi ibrutinib demonstrated a 90.5% ORR and 79.4% MRR in R/R WM, with 5-year OS 87% and PFS 54% (34).

Subsequent iterations of cBKTis include next-generation agents Zanubrutinib and Acalabrutinib. Ibrutinib and Zanubrutinib have received Food and Drug Administration and European Medicines Agency approvals for use in WM, whereas Acalabrutinib is not available in Europe for this indication. There was no statistically significant difference in response rates between Ibrutinib and Zanubrutinib in the randomised phase III clinical trial ASPEN (94% vs. 95%, respectively) for R/R WM, with a median duration of response not reached at 44 months follow-up. The observed side effect profile was generally more favourable with Zanubrutinib (35). Acalabrutinib demonstrated efficacy in WM in a single-arm phase II study, with a 93% ORR of 93% both frontline and in R/R WM. cBTKi orelabrutinib and tirabrutinib have also demonstrated efficacy in Chinese and Japanese multicentre studies (36, 37).

However, complete responses are not attained with cBTKi and drug discontinuation often leads to rapid disease flare, necessitating continuous therapy until progression (38). Disease progression and resistance to cBTKi occur through several mechanisms. Half of cases due to the subclonal mutation BTK^C481S^, affecting the drug binding site, as well mutations in the downstream protein PLCγ2 (39). Patients who are quadruple-agent refractory (i.e., to an alkylating agent, rituximab, cBKTi, and PI) have a poor prognosis, with a median OS of 13.2 months (38).

Novel targeted agents under investigation in clinical trials include BCL2 inhibitors (BCL2i), non-covalent BTKi (ncBTKi) [such as nemtabrutinib and pritobrutinib (40, 41)], BTK degraders, and cellular therapies with bispecific T-cell engagers (such as epcoritamab) and chimeric antigen receptor T-cell (CAR-T) therapy (42, 43).

This review will focus on the role of BCL2 inhibitors.

## 4 Rationale for BCL2 inhibitor use in LPL/WM

The B-cell lymphoma 2 (Bcl-2) family of proteins has long been known to be key cellular apoptotic regulators (44), and the relationship between its pro- and anti-apoptotic members largely determines whether a cell survives or dies (45). Bcl-2 is the name of one of the constituent anti-apoptotic, pro-survival proteins within the wider Bcl2-group, which is capable of sequestering the pro-apoptotic proteins BAK, BAX, and other Bcl-2 homology domain 3(BH3)–only proteins. The regulation of Bcl-2 family proteins is complex but ultimately results in a binary decision whether a cell lives or undergoes apoptosis.

The therapeutic use of BCL2i has achieved impressive results in other B-cell disorders, notably chronic lymphocytic leukaemia (CLL), and drastically shifted the treatment landscape away from CIT (46). Gene expression profiling in LPL/WM cells has identified upregulation of BCL2, similar to that seen in CLL (47). *In-vitro* exposure of *MYD88^MUT^* cells, both *CXCR^WT^ and CXCR^WHIM^*, resulted in apoptosis and has paved the way for therapeutic studies (47).

## 5 Existing evidence for BCL2 inhibitors in LPL/WM

No BCL2i is currently licenced for the treatment of WM/LPL. The only licensed BCL2i in haematological disorders is venetoclax, a small molecule that binds to BH3 and prevents the sequestering of anti-apoptotic proteins, thereby promoting apoptosis (48). It is licenced for and widely used in, CLL and acute myeloid leukaemia (AML). Several other BCL2 inhibitors are in development. Sonrotoclax is a second-generation, highly potent, and selective inhibitor of BCL2. It has shown greater *in vitro* inhibition of BCL2 than venetoclax, and currently undergoing testing in clinical trials. Interestingly, it has shown the ability to overcome several common venetoclax-resistance mutations, such as G101V in pre-clinical mouse models (49, 50).

To date, five studies have evaluated the use of BCL2i in LPL/WM (**Table 1**):

- The first was a phase 1/2 multicentre dose-escalation trial (NCT00062244), by Gertz and colleagues at the Mayo clinic, in patients with WM who received oblimersen sodium. This was an antisense oligonucleotide for the first six codons of the BCL2 open reading frame, which prevents the expression of the gene product. The results of the phase 1 portion were published in 2005, with a partial response in one of nine enrolled patients, but with grade 3 or higher haematological toxicities in five patients (51). A total of 58 patients were enrolled between 2003 and 2007, but no formal results of the phase 2 portion have been published to date (52).
- A phase I dose-escalation study (NCT01328626) by Davids et al. sought to evaluate the safety, pharmacokinetics, and preliminary efficacy of venetoclax in patients with R/R non-Hodgkin lymphomas. Venetoclax was given once daily until progressive disease or unacceptable toxicity, with stepwise dose titration to a maximal dose of 200–1200 mg. Treatment was given on a 3-week dose escalation protocol in most patients. The study began recruiting in 2011 and primary completion occurred in 2020. A total of 106 patients were enrolled, but only four patients with WM. The median age of WM patients was 67 years (range: 58–73 years), with a median of four prior lines of therapy. ORR and MRR were 100%, with all four patients attaining a partial response (PR). Median time to first response was 2.6 months and median duration of response 25.3 months. No patient with WM experienced tumour lysis syndrome (TLS). Study-wide rates of haematological toxicity were <20%, with 49% patients experiencing nausea, 46% diarrhoea, and 44% fatigue (53).
- A multicentre, prospective phase II study of fixed-duration venetoclax monotherapy in patients with previously treated WM (NCT02677324), by Castillo et al., recruited between 2016 and 2018. A total of 32 patients with WM were recruited, with a median of one prior treatment line. Sixteen patients had received previous cBTKi and seven were cBTKi-refractory. All patients harboured MYD88^L265P^ and 17/32 CXCR4^WHIM^ mutations. Venetoclax was administered orally once daily at 200 mg for 1 week, followed by 400 mg for 1 week, and then 800 mg for a total of 24 months. A study amendment allowed venetoclax to be administered orally once daily at 400 mg for one week followed by 800 mg for 24 months. ORR was 84% and MRR 81%. Categorical responses included 19% very good partial response (VGPR), 61% PR, and 3% minor response (MR). No patient attained a CR. Median PFS was 30 months, with 12- and 24-month PFS rates were 83% and 80%, respectively. Six patients progressed within the first 24 months, and 13 after completion of 24 months of venetoclax therapy. Prior cBTKi exposure was associated with longer duration to response but did not affect the maximum depth of response or PFS. All 32 patients were alive at time of data cutoff, with a 30-month OS rate of 100%. One patient experienced biochemical TLS, in the context of moderate nodal disease, splenomegaly, and peripheral lymphocytosis. Neutropaenia was common, including 14/32 grade ≥3 neutropaenias (54).
- A multicentre, single-arm prospective phase II study evaluated ibrutinib and venetoclax combination therapy in patients with previously untreated WM for a fixed duration of 24 months, by Castillo et al. (NCT04273139) (16). A total of 45 patients were recruited between 2020 and 2022. MYD88^WT^ patients were excluded. Treatment cycles were administered every 28 days. Cycle one consisted of ibrutinib 420 mg, with the addition of venetoclax from cycle 2. Venetoclax was administered once daily at 100 mg for 1 week, 200 mg for another week, and 400 mg for 2 weeks. From cycles 3 to 24, participants received ibrutinib 420 mg and venetoclax 400 mg once daily, unless there was disease progression or unacceptable toxicity. TLS prophylaxis was provided in the form of outpatient oral hydration and allopurinol. The ORR was 100% and MRR 96%, with 42% attaining a VGPR, 53% PR, and 4% MR. The 24-month OS rate was 96% and PFS 76%. Crucially, ventricular arrhythmias occurred in three patients, with two deaths and one grade 4 event. The affected patients were all male, >65 years, and had cardiac comorbidities including a history of arrhythmia, coronary artery disease, hypertension, hypercholesterolemia, diabetes, or obesity. The study therapy was subsequently terminated after a grade 2 ventricular arrhythmia occurred in a participant undergoing a precautionary cardiac stress test, resulting in a concerning overall rate of ventricular arrhythmias of 9% in this study. Neutropaenia was common, including 17/45 grade ≥3 neutropaenias. The study authors concluded that there is a likely additive effect of combining BTK and BCL2 inhibitors in WM, with high rates of VGPR, rapid responses, and significant reduction in bone marrow burden (decreasing from 60% to 5% at best response), but that there was an unacceptably high incidence of ventricular events, including two deaths. Intriguingly, such events were not seen in other trials using ibrutinib and venetoclax combinations for other B-cell neoplasms (55, 56). Possible explanations include undetected cardiac involvement by AL amyloid, other cardiac paraprotein deposition, or an inherently higher risk of cardiac events in the patient cohort due to co-morbidities. The combination of ibrutinib and venetoclax for WM/LPL is therefore not recommended or currently planned for further study (16).
- A phase 1a/1b open-label dose escalation and expansion study is examining the use of second-generation sonrotoclax in patients with mature B-cell malignancies (NCT04277637) (57). Interim results were reported on a dedicated cohort of 17 patients with R/R WM, enrolled into three dose escalation cohorts. Previous cBTKi-exposure was noted in 10 patients. At a median follow-up of 10.6 months, four (24%) patients had progressed and two had discontinued due to adverse events. ORR was 76%, MRR 41%, and VGPR 12%. The study aims to complete by 2027.

**Table 1:** Existing trials of BCL2 inhibition in WL/LPL.

ClinicalTrials.gov	Year	Regimen	Phase	Population	Results
NCT00062244	2003–2007	Oblimersen Sodium	I/II	9 R/R	ORR 11%, MRR 11%, and PR 11%
NCT01328626	2011–2020	Venetoclax 200–1200 mg	I	4 R/R	ORR 100%, MRR 100%, and PR 100% Median DOR 25.3 months
NCT02677324	2016–2018	Venetoclax 800 mg 24-month fixed duration	II	32 R/R	ORR 84%, MRR 81% MR 4%, PR 61%, and VGPR 19%
NCT04273139	2020–2022	Venetoclax 400 mg + Ibrutinib 420 mg	II	45 R/R	ORR 100%, MRR 96%, VGPR 42%, PR 53%, MR 4%, OS 96%, and PFS 76% at 24 m
NCT04277637	2020-	Sonrotoclax	I	17 R/R	ORR 76%, MRR 41%, and VGPR 12%

## 6 Trials in progress for BCL2 inhibitors in LPL/WM

There are currently three trials in progress, evaluating the use of BCLi in LPL/WM (**Table 2**):

- A multicentre randomised phase II study is testing the combination of fixed-duration venetoclax and rituximab versus ibrutinib and rituximab for treatment naïve WM/LPL, given for a fixed-duration of 24 months (NCT04840602). The study is led by Dr. Sikander Ailawadhi at the SWOG Cancer Research Network. Enrolment commenced in 2022, with the aim to recruit 92 patients in total. Study completion is estimated by 2028. The primary objective is to compare the rates of ≥VGPR, with secondary objectives including the ORR, PFS, and OS rates (58).
- A single-arm open-label phase II study is evaluating the safety and efficacy of venetoclax in combination with the ncBTKi pirtobrutinib in R/R WM, for a total duration of 24 months (NCT05734495). The study is led by Dr. Jorge Castillo at the Dana Farber and began recruiting in 2023, aiming to enrol 42 patients. Study completion is estimated by 2033. The primary outcome measure is the incidence of ≥VGPR, with secondary objectives including PFS and OS rates (59).
- A multi-arm open label phase II study is evaluating the efficacy and safety of sonrotoclax in patients with WM who are refractory or intolerant to cBKTi (NCT05952037). It seeks to enrol a total of 105, with study completion by 2028. There are three cohorts: cohort 1 is for participants with R/R disease to both cBTKi and anti-CD20–containing CIT, cohort 2 R/R disease to anti-CD20–containing CIT with intolerance to BTKi, and cohort 3 R/R disease to BTKi and unsuitable for CIT. In addition, a fourth subcohort will evaluate the use of sonrotoclax in combination with cBTKi zanubrutinib in treatment-naïve patients for a fixed duration. The primary outcome measure is MRR, with secondary outcomes including ORR, rates of ≥VGPR, duration of response, PFS, and OS (60).

**Table 2:** Trials in progress of BCL2 inhibition in WL/LPL.

ClinicalTrials.gov	Year	Regimen	Phase	Population	Outcome measures
NCT04840602	2022-	Venetoclax-Rituximab versus Ibrutinib-Rituximab 24-m fixed-duration	Randomised II	Naïve	1°: Rates of ≥VGPR in each arm 2°: ORR, PFS and OS, safety
NCT05734495	2023-	Venetoclax-Pirtobrutinib 24-m fixed-duration	Single arm II	R/R	1°: Rates of ≥VGPR 2°: Best response, PFS, time to next treatment (TTNT), DOR, OS, quality of life (QoL)
NCT05952037	2023-	Cohort 1-3: Sonrotoclax Cohort 4: Sonrotoclax-Zanubrutinib	Multiarm II	Cohorts 1-3: R/R Cohort 4: Naive	1°: Rates of ≥MRR 2°: ORR, ≥VGPR, DOR, PFS, OS, TTNT, QoL

## 7 Conclusion

WM/LPL is an indolent but incurable condition, typically requiring several lines of therapy punctuated by periods of relative remission. Although CIT continues to be used, particularly for treatment-naïve patients where it has demonstrated good response rates and PFS, cBTKis are the mainstay of R/R WM. With increasing duration of use, resistance to cBTKis is an emerging problem and other oral agents are needed. A proportion of patients are also unable to tolerate BTKis due to class-specific side effects, such as bleeding. In addition, the prospect of a “chemotherapy-free,” fixed-duration frontline therapy would no doubt be appealing to patients and clinicians.

A total of five studies have examined the use of BCL2 inhibitors in WM/LPL: one oblimersen sodium, three venetoclax, and one sonrotoclax. Three pivotal trials are currently in progress, with the potential to alter practice in the treatment of LPL/WM. Fixed-duration venetoclax monotherapy for 24 months resulted in high-response rates but significant progression within 12 months of completion. This suggests that either continuous or combination therapy is required for the use of BCL2i in this condition. The combination of a BTKi ibrutinib and BCL2i venetoclax in LPL/WM, unfortunately, resulted in unacceptably high rates of ventricular events and death (16), essentially excluding this drug combination from further evaluation in WM/LPL. A cautious interpretation of the rapid responses and high rates of VGPR with this combination suggests that BCL2i in combination with other covalent- and non-covalent BTKis, with less cardiotoxic side effect profiles and in selected patients, may be of interest. The combination may particularly have a role in the context of heavy bone marrow infiltration, given the high rates of bone marrow clearance seen. This combination is currently being evaluated in two studies, one using sonrotoclax-zanubrutinib frontline and another using venetoclax-pirtobrutinib for R/R WM. The combination of a BCL2i with an anti-CD20 antibody, such as rituximab, may also prove efficacious.

The role of BCL2 inhibitors in WM/LPL remains under study, with many hopeful that they may provide an additional chemotherapy-free oral alternative for patients requiring treatment, both frontline and in the R/R setting. In an indolent condition with existing effective options, including CIT and cBTKi, cost-effectiveness and toxicity profile will be key. However, they are likely to find an important role if capable of offering an effective therapeutic mechanism for patients’ refractory to existing therapeutic options with limited options, particularly quadruple refractory patients.
